# Fruit Detection and Segmentation for Apple Harvesting Using Visual Sensor in Orchards

**DOI:** 10.3390/s19204599

**Published:** 2019-10-22

**Authors:** Hanwen Kang, Chao Chen

**Affiliations:** Laboratory of Motion Generation and Analysis, Faculty of Engineering, Monash University, Clayton, VIC 3800, Australia; hanwen.kang@monash.edu

**Keywords:** deep learning, machine vision, real-time fruit detection, semantic segmentation, visual sensor, automated harvesting robot

## Abstract

Autonomous harvesting shows a promising prospect in the future development of the agriculture industry, while the vision system is one of the most challenging components in the autonomous harvesting technologies. This work proposes a multi-function network to perform the real-time detection and semantic segmentation of apples and branches in orchard environments by using the visual sensor. The developed detection and segmentation network utilises the atrous spatial pyramid pooling and the gate feature pyramid network to enhance feature extraction ability of the network. To improve the real-time computation performance of the network model, a lightweight backbone network based on the residual network architecture is developed. From the experimental results, the detection and segmentation network with ResNet-101 backbone outperformed on the detection and segmentation tasks, achieving an $F_{1}$ score of 0.832 on the detection of apples and 87.6% and 77.2% on the semantic segmentation of apples and branches, respectively. The network model with lightweight backbone showed the best computation efficiency in the results. It achieved an $F_{1}$ score of 0.827 on the detection of apples and 86.5% and 75.7% on the segmentation of apples and branches, respectively. The weights size and computation time of the network model with lightweight backbone were 12.8 M and 32 ms, respectively. The experimental results show that the detection and segmentation network can effectively perform the real-time detection and segmentation of apples and branches in orchards.

## 1. Introduction

Apple harvesting is a labour-intensive, time-consuming, and costly task. The ageing population and cost of the human resources has led to a decreasing of available labour force for the agriculture harvesting [1]. Therefore, automatic harvesting robots that can automatically work in the field are becoming a promising technology in the future development of the agriculture industry. Different from the traditional automatic harvesting of crops, automatic harvesting of fruits such as apple is in a more complicated case [2]. Robotic harvesting of fruit requires the vision system to detect and localise the fruit. Furthermore, to increase the success rate and reduce the damage rate of automatic fruit harvesting, information of the fruit pose [3] and stem–branch joint location and orientation [4] are also required. This demands the robotic vision system to accurately and robustly extract the geometry and semantic information from the working scene in the orchard environment [5]. Recently, with the advancements of the depth camera technologies, the harvesting robotic vision system is able to model and present the working scene in the three-dimensional form [6]. However, it is still challenging to robustly and accurately perform semantic processing of the visual data in the orchard environment, such as detection and segmentation of the fruit and branch, due to various factors such as illumination variance, occlusion, and variations of object appearance. To overcome these, it is crucial to develop a highly effective and robust vision algorithm for the fully automatic harvesting [3]. In this work, a multi-function Deep Convolution Neural Network (DCNN) is developed to perform the real-time detection and semantic segmentation of apples and branches in orchards. Firstly, to enhance the feature extraction ability of the network, the Gated Feature Pyramid Network (GFPN) and the Atrous Spatial Pyramid Pooling (ASPP) are utilised in the developed Detection and Segmentation Network (DaSNet). Secondly, to facilitate the fast computation of the network on the embedded computing device, a lightweight network (lw-net) is developed based on the residual network architecture. In the experiment, we evaluated the performance and efficiency of the DaSNet with different backbones and different detector architectures. The comparison between the DaSNet and other deep-learning based detection and segmentation works was also included.

The rest of the paper is organised as follows. Section 2 reviews the related work of fruit detection techniques. Section 3 introduces the DaSNet model in detail. The experiment and discussion are demonstrated and analysed in Section 4 and Section 5 concludes the work.

## 2. Related Work

Fruit detection has been studied extensively in the past few decades. Several kinds of sensors have been applied in the automation of fruit harvesting [7], including RGB/RGB-D camera, laser sensor, thermal imaging sensor, and spectral imaging sensor. This work focuses on reviewing the techniques which are developed for RGB image processing. Imaging detection can be classified into two groups: conventional machine-learning based algorithms and deep-learning based algorithms. The former methods use the image feature descriptors to encode the feature information, and then apply the machine-learning based classifier to perform the segmentation or detection the fruit within the image [8]. There are many expert-coded feature descriptors that have been developed, such as the histogram of gradient [9], the colour coherence vector [10], and the local binary patterns [11]. Similarly, many machine-learning based classifiers have been developed, such as the clustering, the Support Vector Machine (SVM) and the neural network. Traditional machine-learning based algorithms have been widely applied in automatic agriculture applications. Zhou et al. [12] proposed a colour feature-based logistic regression classifier to detect apples in the orchard environment. Song et al. [13] applied a Bayes classifier and SVM to learn the colour and texture features, in order to detect peppers with an RGB camera. Luo et al. [14] and Wang et al. [15] utilised the colour feature-based and texture-feature based AdaBoost classifier to perform the fruit detection.

Recently, DCNN shows promising performance in many computer vision tasks, including object classification [16], object detection [17], and image segmentation [18]. Compared to traditional machine-learning based algorithms, DCNN achieves a more robust and accurate performance due to its strong feature extraction ability and autonomous learning mechanism [19]. There are two kinds of DCNN model that have been developed to perform object detection: two-stage detectors and one-stage detectors. Region Convolution Neural Network (RCNN) is one of the most successful works of the two-stage detector, including RCNN [20], Fast/Faster RCNN [21,22], and Mask RCNN [23]. RCNN contains two sub-tasks networks: the Region Proposal Network (RPN) and the classification network. The RPN searches the location of Region of Interest (ROI), while the classification network predicts the class of ROI and regresses the boundary box of the ROI candidates. RCNN has been widely applied in many vision-guided automatic agriculture applications. Sa et al. [24] applied Faster RCNN on multi-vision sensor to detect peppers, rock-melons and apples. Bargoti and Underwood [25] adopted the faster-RCNN model on the detection of apples and mangos in orchards. Yu et al. [26] applied the mask RCNN to perform the detection and segmentation of the strawberry in the greenhouse, in order to guide the automatic harvesting of strawberries. Another DCNN model, called “one-stage detector” was developed more recently. Representative methods of one-stage detector are Single Shot Detection (SSD) [27,28] and You Only Look Once (YOLO) [29,30,31]. The one-stage detector combines RPN and classification network into a single architecture, which largely reduces the computation cost of the forward inference. The one-stage detector has been gradually studied and applied in the vision-guided automatic agriculture applications such as the yield estimation and automatic harvesting. Tian et al. [32] modified an improved YOLO-V3 network to perform real-time detection of apples, which is developed to monitor and evaluate the growing of apples in orchards. Koirala et al. [33] applied a lightweight YOLO network to perform the yield estimation of mangos in orchards, and reported an $F_{1}$ score of 0.89 in their work.

Semantic segmentation is another essential computer vision task, which predicts a class label in each of pixel of the image [18]. Compared to object classification task, which predicts the image class while losing the spatial information of the objects, semantic segmentation can preserve the spatial information of the objects and predict its shape within the image [34]. Typical deep-learning based semantic segmentation network applies the auto-encoder architecture to encode the image data and generate the semantic segmentation of the image. Rather than using the sliding windows strategies to classify all pixels within the image [35], deep-learning based semantic segmentation network can predict the labels for all pixels within the image in a single forward inference. Many semantic segmentation network architectures have been developed for different applications, such as the Full Convolution Network (FCN) [36], SegNet [37], and the DeepLab [38,39,40], which are designed for general applications, and Unet and Vnet [34,41], which are designed for medical image analysis. Semantic segmentation network has also been applied in many agriculture applications. McCool et al. [42] developed a multi-feature classifier to perform the semantic segmentation on peppers, which is used to guide the robotic harvesting of peppers. Bargoti and Underwood [43] developed a multi-Layered Perception to segment apples for yield estimation in orchards. Li et al. [44] applied an FCN model to perform the automatic ground-based in-field cotton segmentation. Lin et al. [5] applied an FCN model to perform the segmentation of guava fruits and branches and estimate the pose of guava fruits based on the segmented information to guide the robotic harvesting. It was reported that FCN model achieved higher segmentation accuracy than traditional algorithms.

## 3. Material and Methods

### 3.1. Vision Sensing System

**Figure 1:**
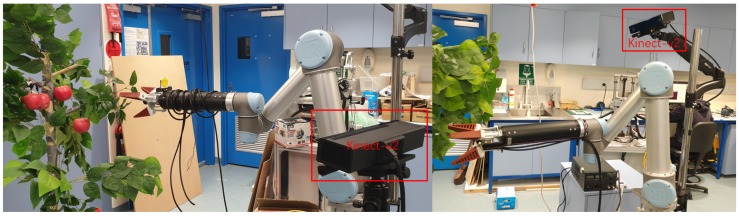
The developing apple harvesting robot. The robot comprises a RGB-D camera for vision sensing. A universal robot arm (UR5) is applied as the manipulator.

The developed apple harvesting robot includes a UR-5 robotic arm and an RGB-D visual sensor, as shown in Figure 1. The RGB-D camera applied in this work is the Kinect-v2, which is developed by Microsoft Inc. The Kinect-v2 is comprised of an RGB camera and an infrared(IR) depth camera, and can capture colour images with the resolution in the range between 640 × 480 and 1920 × 1080. The IR depth camera of the Kinect-v2 can capture depth images with the resolution of 424 × 512. During working, the depth image is resized to be consistent with the colour image size and fused. Based on the previous in-field experiments and the computation ability of the applied embedded computing device, the resolution of 640 × 480 is used in this work. In the experiment, the Kinect-v2 was controlled using the ROS-kinetic in Ubuntu 16.04 with the libfreenet2 SDK tool.

### 3.2. Network Architecture

#### 3.2.1. Gated-FPN for Multi-level Fusion

**Figure 2:**
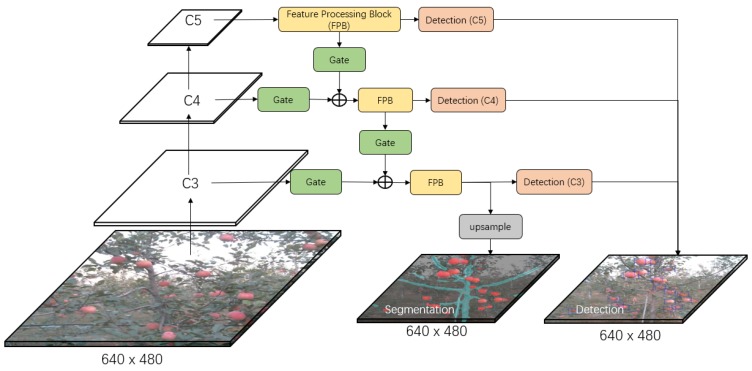
The architecture of DaSNet-A model. DaSNet-A has the common GFPN and the feature processing block for processing of detection and segmentation tasks. The output of the feature processing block in C3 level is upsampled to the original image size to generate the semantic segmentation prediction.

**Figure 3:**
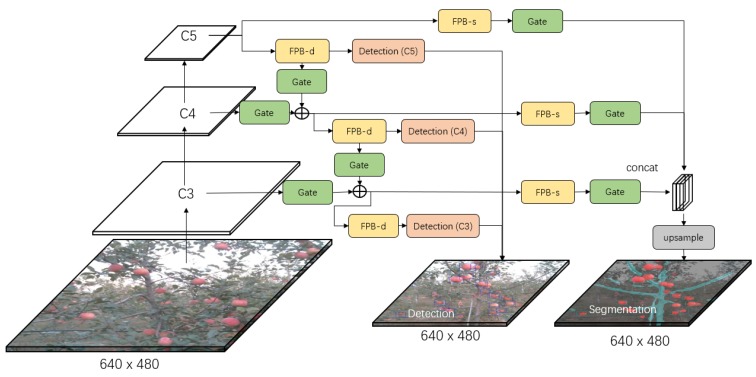
The architecture of DaSNet-B model. DaSNet-B has the common GFPN but the independent feature processing block for processing of detection and segmentation tasks. FPB-s stands for the feature processing block of the segmentation branch, while FPB-d stands for the feature processing block of the detection branch.

**Figure 4:**
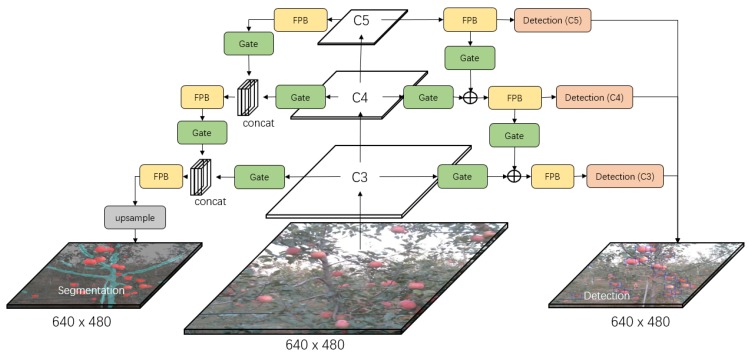
The architecture of DaSNet-C model. DaSNet-C has the independent GFPN and the feature processing block for processing of detection and segmentation tasks separately.

**Figure 5:**
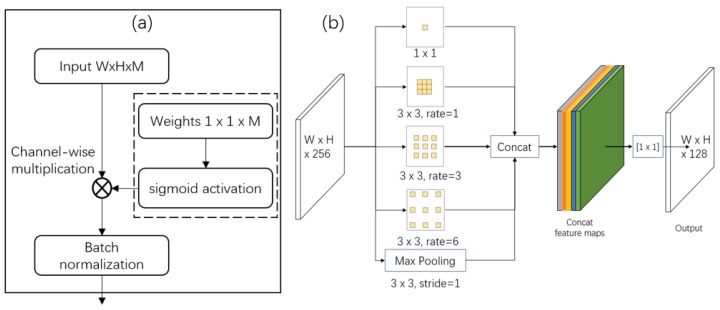
(**a**) The architecture of gate in the GFPN; and (**b**) the architecture of ASPP in the feature processing block.

Figure 2, Figure 3 and Figure 4 show the three developed architectures of the DaSNet, which are named DaSNet-A, DaSNet-B and DaSNet-C, respectively. DaSNet adopts multi-level feature pyramid network to receive the feature tensors from the C3 layer (1/8), C4 layer (1/16) and C5 layer (1/32) of the backbone network. From the previous study of the representation of the feature in the DCNN model, features in different levels of the network contain different information of the objects [45]. The lower level network (such as C3) mainly includes the spatial information of the objects, while the higher level network (such as C5) mainly includes the semantic information of the objects. A recent study by Yao et al. [46] has pointed out that direct fusion of the different levels of the network can lead to the spatial shift of the feature and unbalance gradient propagation in the network training. Therefore, they developed Gated-FPN to minimise the effect of the above issues. Similarly, GFPN design is adopted in the DaSNet architecture to enhance the feature expression of the model. The GFPN design adopted in the DaSNet is inspired by the work of the Long Short-term Memory (LSTM) [47] and the Gated Recurrent Unit (GRU) [48], which adopt gate network to enable the network to selectively memorise or forget the information within the sequences data. The GFPN in DaSNet adopts a channel-wise multiplication on each channel of the input feature tensors, to allow the network to adjust the weights of the feature in the feature maps. The weights used for the channel-wise multiplication is pre-activated by the sigmoid function, to allocate the value range of the weights from zero to one. A batch-normalisation layer is added after the gate as our experiment shows that the batch-normalisation layer can improve the performance of the network model. The GFPN in the DaSNet allows the selective representation of the feature between different levels, which can minimise the spatial shift of the feature maps from different levels and balance the backward propagated gradients. The architecture of the GFPN used in DaSNet is shown in Figure 5.

Three different DaSNet architectures are developed in this work, as shown in Figure 2, Figure 3 and Figure 4, respectively. The details about these three network architectures and its correspond training methods are included in Section 4.1.

#### 3.2.2. ASPP for Multi-Scale Fusion

**Figure 6:**
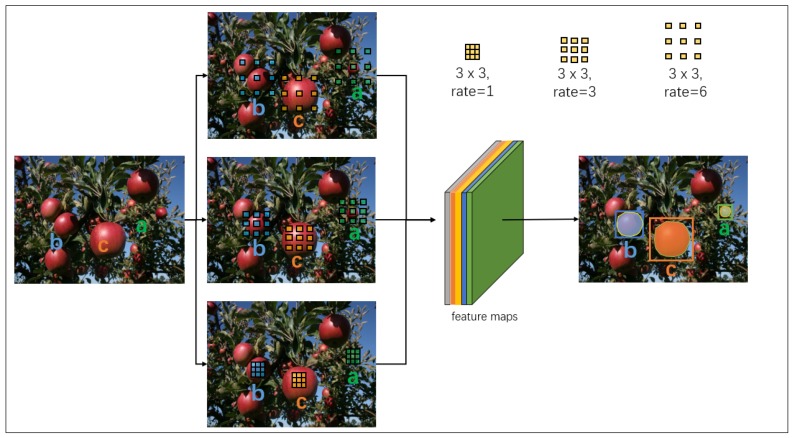
The ASPP adopts the dilation convolution with different dilation rates to encode the multi-scale information of objects.

Each level of the GFPN in the DaSNet adopts a feature processing block to process the feature maps before it is fed into the detection and segmentation branch. DaSNet utilises the ASPP to enhance the feature extraction of the multi-scale information of the objects; the architecture of the feature processing block is shown in Figure 5. The ASPP has been applied in many previous works on object detection [46] and segmentation [49]. It relies on the dilation convolution with different dilation rate to encode the multi-scale information of the objects into a single pixel within the feature maps, as shown in Figure 6. The ASPP adopted in the DaSNet applies three branches, which include three 3 × 3 dilation convolutions with dilation rates as 1, 3 and 6, and the feature maps on each branch of the ASPP have 64 channels. In addition, another branch, which adopts a 3 × 3 max-pooling layer, is also applied in the ASPP, as our experiment suggested that max-pooling can improve the detection and segmentation performance of the model. To keep the consistency of the channel number of the feature maps on each branch of the ASPP, a convolution layer with a 1 × 1 kernel is adopted after the max-pooling. All the branches of the ASPP are concatenated to generate the combined feature map. Then, a 1 × 1 convolution layer is applied to fuse the information within the combined feature map and reduce the channel number of the feature maps to 128.

#### 3.2.3. Lightweight Designed Backbone

**Figure 7:**
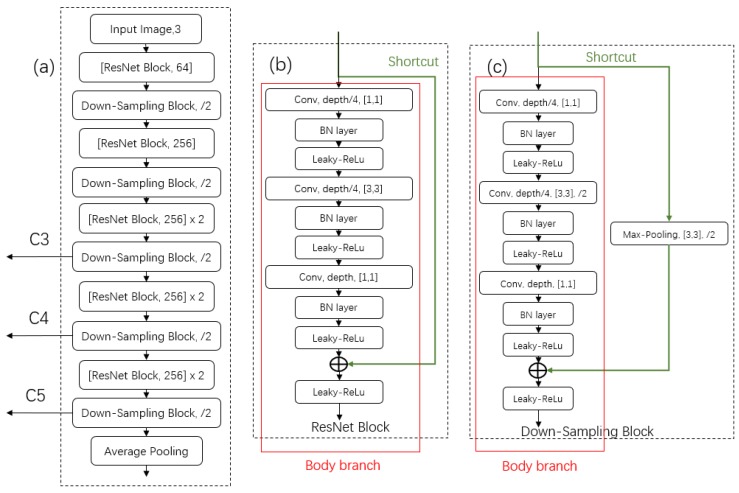
The architecture of the lw-net: (**a**) the lw-net architecture; and (**b**,**c**) the bottleneck residual network block and down-sampling block architectures, respectively.

To reduce the computing cost and facilitate the real-time applications of the DaSNet model in the embedded computing device, a lightweight backbone network (lw-net0 is developed based on the residual network architecture [50]. The lw-net adopts the bottleneck residual network block design to reduce the weight size and computation complexity of the network inference. Meanwhile, to reduce the feature information loss during the under-sampled pooling operation, the max-pooling layer of the original residual network architecture is replaced with a modified down-sampling block design. Both the bottleneck residual network block and the down-sampling block comprise two branches: the body branch and the shortcut branch, which are shown in Figure 7. The bottleneck residual network block design shortcuts to add the input feature map from the input of the block to the output of the block. The down-sampling block applies a max-pooling layer in the shortcut branch to perform the pooling of the input feature maps. Meanwhile, the second convolution layer in the body branch of the down-sampling block applies a convolution layer with stride 2 to perform the pooling of the feature maps as well. The lw-net architecture includes nine bottleneck residual network blocks and five down-sampling blocks. In the experiment, the lw-net was pre-trained on the Cifar-10 and the Cifar-100 datasets. The validation accuracies of the lw-net on the Cifar-10 and the Cifar-100 datasets were 92.7% and 71.6%, respectively. The size of the total weight of the lw-net is only 3.46 MB. In the experiment, the lw-net was further trained with the collected orchard image data, with the resolution of the training image set to 128 × 128 and the object classes were apple, branch and background. In addition to the developed lw-net, for acceleration of network computation purpose, some other state-of-the-art classification networks were also applied as the backbone for the DasNet model. In the experiment, ResNet-50/ResNet-101 [50] and Darknet-53 [31], which were pre-trained with ImageNet dataset, were applied as the backbone network of the DasNet model.

### 3.3. Training Data and Method

#### 3.3.1. Data Collection

The data were collected by using the Kinect-v2 in the orchard located in Qingdao, China. The collection time of the image data was from 08:00 to 18:00. We collected 800 images from the orchards in total. The ground truth of the object detection was labelled by using the “LabelImg”, which is publicly available on Github. The ground truth of the semantic segmentation was labelled by using the windows drawing tool and surface pen. In the following experiment, 600 out of 800 images were used to train the network, while the other images served as the validation data.

#### 3.3.2. Training Method

**Figure 8:**
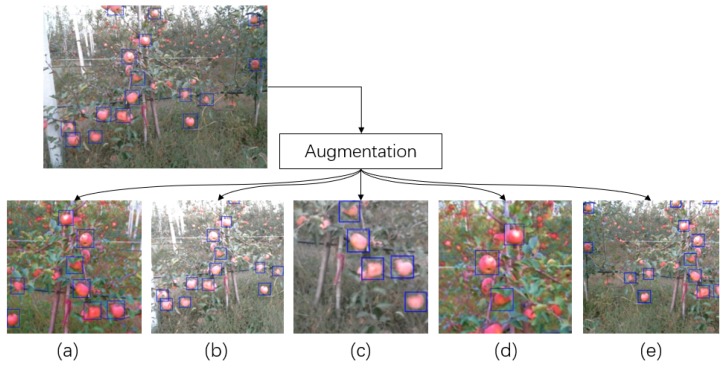
Examples of augmentation method applied in the network training using: (**a**) crop, flip and saturation adjustment; (**b**) brightness and contrast adjustment; (**c**) crop and flip; (**d**) crop and saturation; and (**e**) flip.

The data were collected from a mobile vehicle that works in the orchard. The distance between apple trees to the vehicle was between 0.8 and 1.5 m, which is also the working distance of the developed apple harvesting robot. The diameter of apples in the orchard were 80–100 mm. At the working distance of 0.8–1.5 m, most apples in the training data were presented in the form of small scale objects. This unbalanced distribution of the scale of the objects may lead to under-fitting issue during the training of the anchor-box based detector. Therefore, an augmentation method that can minimise the unbalanced distribution of the scale of the objects within the training data was utilised in this work. The resolution of the original image in the training dataset is 640 × 480, while the resolution of the image used for the network training is 320 × 320. The reason for applying 320 × 320 as the training resolution is to increase the number of the image in each training batch, which can stabilise the training process of the batch normalisation layer [51]. During the training, the augmentation algorithm had the probability of 0.5 to crop a patch whose size is between 160 × 160 and 320 × 320 from the original image and resize the cropped patch to the training resolution. Then, this step had the probability of 0.5 to be repeated another time to further amplify the small objects within the training data. Other image augmentations, including image flip, colour saturation, contrast and brightness adjustment, and translation, were also applied during the training. Several examples of the training data that were processed by the applied augmentation are shown in Figure 8. To analyse the distribution of the scale of the objects within the training data before and after the augmentation process, a statistical analysis was performed, which is shown in Table 1.

From the statistical result of the training data shown above, the applied augmentation algorithm can minimise the unbalanced distribution of the scale of the objects in the training data. However, considering the number of objects in the large scale in the training data is still limited compared to the objects in the small and medium scale in the training data, some open-source image data were collected into the training dataset to further balance the distribution of the scale of the objects. During the training, the Adam-optimiser was used to train the DasNet; the learning rate and decay rate used in training were 0.01 and 0.9/epoch based on our previous experiment results.

## 4. Experiment and Discussion

The DaSNet code was implemented in Tensorflow 1.11 and trained on the Nvidia GTX-1080Ti. The Kinect-v2 was controlled using the ROS-kinetic on the Ubuntu 16.04. The pre-trained weight and implement code of ResNet-50/101 in Tensorflow [52], YOLO-V3/YOLO-V3(tiny) in tensorflow [53], Faster-RCNN in caffe [54], and FCN-8s(ResNet-50/ResNet-101) in Tensorflow [55] were from the Github publicly code library.

Mean Intersection of Union (MIoU) [56] was used to evaluate the performance of the network on semantic segmentation. The Average Precision ($AP_{IoU}$) [57], and $F_{1}$ score [58] were used to evaluate the performance of the network on object detection.

### 4.1. Experiment on Network Architecture and Training

#### 4.1.1. Experiment on Network Architectures

DaSNet is developed to perform the detection and semantic segmentation of the multi-class objects within a single network architecture. The object detection task includes the prediction of the confidence score, the boundary box, and the class of the objects, while the semantic segmentation only includes the classification on each pixel within the feature maps. Meanwhile, object detection predicts the objects from the separate level of GFPN (C3, C4, and C5), while the prediction of the semantic segmentation is generated from the upsampled feature maps of the C3 level in the GFPN. Therefore, there may be a significant difference in the distribution of the feature maps between the object detection task and semantic segmentation task.

Three architectures were developed to explore the optimal design of the network, which allows the model to fit the feature distribution of different tasks within a single network. These three models are named as DaSNet-A, DaSNet-B and DaSNet-C and shown in Figure 2, Figure 3 and Figure 4, respectively. DaSNet-A has the common GFPN and feature processing blocks for both object detection and semantic segmentation. The prediction of the object detection is generated from the C3, C4 and C5 levels of the GFPN, while the semantic prediction is generated from the upsampled feature maps of the C3 level of the GFPN. DaSNet-B has the common GFPN but the independent feature processing blocks for the object detection and semantic segmentation. DaSNet-C has the independent GFPN and feature processing blocks for the object detection and semantic segmentation.

Two different training strategies were utilised based on the characters of the different network architectures. The first method “M1” is to train the network on the detection and semantic segmentation tasks simultaneously, while the second method “M2” is to train the network on the detection and semantic segmentation tasks separately. DaSNet-A was trained with the M1 method since the object detection and semantic segmentation share the major body of the network model. DaSNet-B and DaSNet-C were trained with M1 and M2 methods to explore which training strategies are optimal for such network architectures. During the training of DaSNet-B and DaSNet-C with the M2 method, the weights of the detection branch and backbone were frozen, and only the weights of the segmentation branch were involved.

#### 4.1.2. Experiment Results and Discussion

The experimental results of the comparison between the different network architectures and training methods are shown in Table 2.

**GFPN** vs. **FPN**: Experiments 4 and 6 showed the performance evaluation of DaSNet-B with GFPN and FPN, respectively. The results show that GFPN improved the $AP_{50}$ from 79.9% to 82.7%, and increased the $F_{1}$ score from 79.2% to 82.1%. Similar results are also shown in the semantic segmentation: DaSNet-B with GFPN had a higher MIoU score than DaSNet-B with the FPN. DaSNet-B with GFPN achieved 0.865 and 0.757 on the segmentation of apples and branches, respectively. DaSNet-B with FPN achieved 0.832 and 0.722 on the segmentation of apples and branches, respectively. The experiment showed that GFPN can increase the performance of the network in both tasks of object detection and semantic segmentation.

**Network Architectures**: Experiments 1–3 showed the performance of DaSNet-A, -B and -C on the detection and segmentation when M1 training strategy was applied. From the experiment results, DaSNet-C outperformed in the comparison of the performance of the three network architectures. DaSNet-C had the independent GFPN and feature processing block for semantic segmentation and object detection, which allowed the network to fit the feature distribution of the different tasks properly. DaSNet-A had the common GFPN and feature processing blocks for both tasks, which limited the ability of the network to fit the feature distribution of the different tasks. From the experiment results, DaSNet-A showed the least efficient performance of the detection and segmentation.

**Training Methods**: Experiments 2–5 compared the performance of the networks when different training methods were applied. Experiments 3 and 5 compared the performance of DaSNet-C trained with M1 and M2 methods. Since DaSNet-C model had the independent branch for object detection and semantic segmentation, similar results are shown in the comparison. Experiments 2 and 4 compared the performance of DaSNet-B with M1 and M2 methods. From the experiment results, the DaSNet-B showed better performance when the M2 training method was applied. The reasons that contribute to the results are summarised as follows. When M1 training method was applied, backward propagation of different feature distribution on the different tasks may lead to the under-fitting of the weights on both tasks. When M2 training method was applied, the training of the segmentation task only focuses on the update of the weight in the segmentation branch. From the experiment results, this measurement improved the training quality and performance of the network.

**Implementation efficiency**: Table 3 shows the weights size and inference time of the DaSNet-A, -B and -C. Considering the aspect of implementation efficiency and performance, DaSNet-B achieved the equal detection (0.827 and 0.821 on $AP_{50}$ and $F_{1}$ score, respectively) and segmentation performance (86.5% and 75.7% for apple and branch segmentation, respectively) compared to DaSNet-C. It kept a similar implementation efficiency compared to DaSNet-A model (weight size is 12.8 M and inference time is 32 ms). Therefore, the DaSNet-B architecture was considered as the best performing candidate in the experiment, and it was applied as the DaSNet in the following experiments.

### 4.2. Experiment on Detection Performance

This experiment compared the performance and implementation efficiency of the DaSNet-B when different backbone networks were applied, including the ResNet-50, ResNet-101, Darknet, and the developed lw-net. Meanwhile, a comparison between the DaSNet and other state-of-the-art works, includeing YOLO-V3, YOLO-V3 (tiny) and Faster RCNN, was also performed. The experimental results are listed in Table 4.

Experiments 1, 5, 6 and 7 compared the performance of the object detection of DaSNet-B (lw-net), YOLO-V3, YOLO-V3 (Tiny) and Faster RCNN. The experimental results show that DaSNet-B (lw-net) outperformed the three other network models. DaSNet-B (lw-net) achieved 82.7% on AP evaluation and 0.821 on $F_{1}$ score. Meanwhile, DaSNet-B model also outperformed in the implementation efficiency. The weights size and inference time of DaSNet-b Model are 12.8 M and 32 ms, respectively. Experiment 6 was the performance evaluation of the tiny version of the YOLO-V3, which optimises YOLO-V3 in terms of the calculation complexity and time efficiency. From the experiment results, DasNet-B with lw-net achieved better performance on detection task, while kept the equal implementation efficiency with the tiny version of YOLO-V3 network. Experiments 1–4 showed the performance evaluation of the DaSNet-B with different backbone, including lw-net, ResNet, and darknet. As shown in the results, DaSNet-B with ResNet-101 backbone performed the best within the test, achieving 83.6% on AP and 0.832 on $F_{1}$ score. The experimental results indicate that the powerful backbone network can increase the performance of the detection network. However, the implementation efficiency of DaSNet-B (ResNet-101) showed a decrease as the backbone increase the computation complexity of the inference. The weights size and inference time of the DaSNet-B (ResNet-101) are 188 M and 72 ms, respectively. During implementation, DaSNet can use different backbone network based on the computation hardware and the design requirement.

### 4.3. Experiment on Segmentation Performance

**Figure 9:**
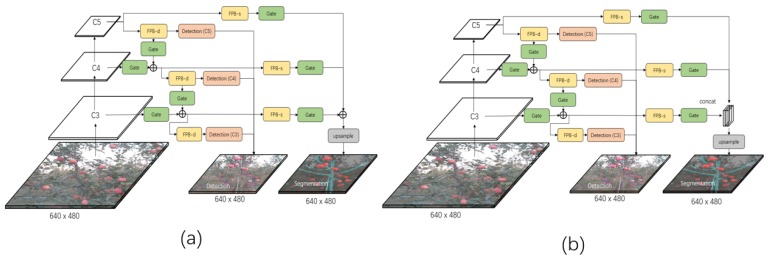
The architecture of DaSNet-B-Add (**a**) and DaSNet-B-concat (**b**). DaSNet-B-Add model uses adding operation to fuse the feature maps for segmentation, while DaSNet-B-Concat model uses concatenate operation.

**Figure 10:**
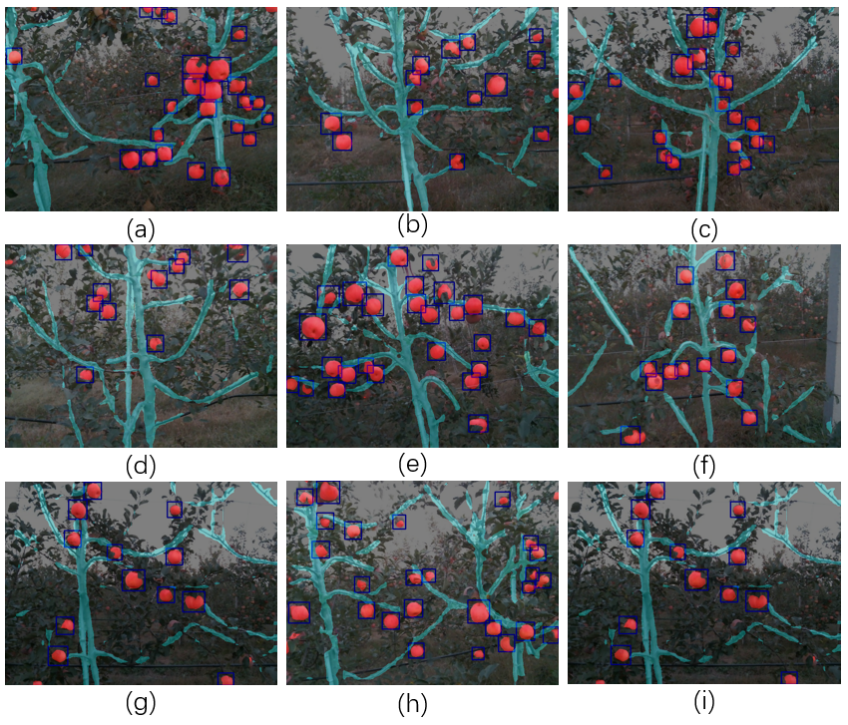
(**a**–**i**) show the segmentation and detection of apples and branches in orchard environment by using the DaSNet.

Semantic segmentation returns a multi-class mask to predict the label of each pixel of the input RGB image, which is a critical task for sensing and understanding the working environment. In this experiment, two different architectures modified from DaSNet-B model were tested. The first model uses the concatenate operation at the C3 layer to fuse the feature maps for semantic label prediction, which is named as the DaSNet-B-Concat model. The second model uses an adding operation to fuse the feature maps for semantic label prediction, which is named as DaSNet-B-Add model. The architecture of these two models are shown in Figure 9. The reason behind this experiment was to explore which operation could generate better performance on the semantic segmentation. Similar to the evaluation of the detection results, several pre-trained backbone networks were applied in this experiment. The FCN-8s network was applied as the baseline algorithm to form the comparison. FCN-8s network uses ResNet-50 and ResNet-101 as the backbone. The FCN-8s network was first trained with PASCAL-VOC2012 and then trained using the orchard data. The results of the experiment are shown in Table 5. The demonstration results of segmentation and detection of apple and branch are shown in Figure 10.

**Figure 11:**
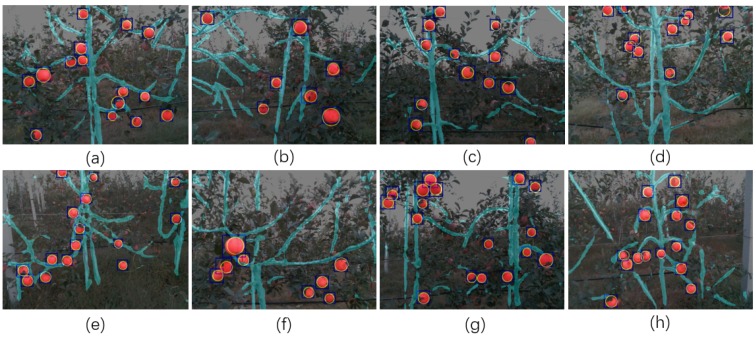
(**a**–**h**) show the instance segmentation (${}^{*}$ yellow circle) of apples by using the circle hough transform based on the detection and segmentation results of the DaSNet.

Experiments 1 and 2 compared the performance of the DaSNet-B-add and the DaSNet-B-concat model. From the experiment results, the DaSNet-B-concat achieved a better MIoU score compared to the DaSNet-B-add model. DaSNet-B-concat achieved 86.5% and 75.7% on the segmentation of apples and branches, i.e., 0.7% and 2.6% higher than the DaSNet-B-add model, respectively. Experiments 2–5 compared the performance of the DaSNet-B-concat model with the different backbone networks. Similar to the experimental results for the detection evaluation, the DaSNet-B-concat model with the ResNet-101 backbone outperformed in the segmentation evaluation. Meanwhile, the DaSNet-B-concat model with the lw-net backbone also showed a good performance on the segmentation of apples and branches, at 86.5% and 75.7%, respectively. Experiments 3, 4, 6, and 7 compared the performance of the semantic segmentation between DaSNet and FCN-8s. From the experiment results, the DaSNet achieved a higher accuracy on the segmentation task compared to the FCN-8s. Especially, the MIoU score of the branch segmentation achieved by the DaSNet was 4.5% and 3.9% higher than the MIoU score achieved by FCN-8s. Since DaSNet adopts ASPP and GFPN to enhance the extraction ability in terms of the multi-scale information and multi-level information, DaSNet achieved a better segmentation performance compared to the FCN-8s model. From the experiment results, the segmentation of the branch is more challenging than the segmentation of apples, as there are many branches which are blocked by the leaves. In this condition, DaSNet could still segment the majority structure of the branch from the background accurately, as shown in Figure 10 and Figure 11.

After the segmentation by using DaSNet, Circle Hough Transform (CHT) was further used to segment apples and its instance mask in each detected boundary box. Several processed image by using the DaSNet and the CHT are shown in Figure 11. The blue box indicates the detected boundary boxes and the yellow circle indicates the detected apple in each of boundary box. With the semantic label and spatial information on each pixel within the image, further processing such as estimation of the fruit pose [5], branch reconstruction [59] and the estimation of the stem–branch joint [3] can be applied accordingly. Such post-processing techniques will be included in future work of our study.

## 5. Conclusions

This work developed a multi-function network DaSNet to perform the real-time detection and semantic segmentation of the apple and branch in the orchards. The DasNet utilises the GFPN to fuse the information from different levels of the model and adopts the ASPP to enhance the feature extraction of multi-scale information of the objects. To improve the real-time computing performance of the model in the embedded computing device, a lightweight backbone network based on the residual network architecture was developed. Based on the different characters of the semantic segmentation and object detection, three different DasNet architectures and corresponding training strategies were developed and evaluated in the experiment. The comparison of the performance between the DasNet and the other state-of-the-art works in object detection and semantic segmentation was included in the experiment. From the results, the DaSNet with ResNet-101 backbone performed the best in both semantic segmentation and object detection tasks. It achieved an $F_{1}$ score of 0.832 on the detection of apples and 87.6% and 77.2% on the segmentation of apples and branches, respectively. The DaSNet with lightweight backbone lw-net achieved a good detection and segmentation performance while it outperformed in the computation efficiency. It achieved an $F_{1}$ score of 0.821 on the detection of apples and 86.8% and 75.7% on the segmentation of apples and branches, respectively. The weights size and inference time of the network model was 12.8 MB and 32 ms, respectively. Overall, the developed DasNet can perform real-time detection and segmentation in the orchards. 

## Figures and Tables

**Table 1:** Statistic of different scale of object in the training data during the network training.

Index	Iteration	If Augmentation	Small	Median	Large
1	100	Yes	67%	32%	1%
2	100	No	87%	13%	0%
3	1K	Yes	58%	37%	5%
4	1K	No	85%	12%	1%
5	10K	Yes	51%	40%	9%
6	10K	No	89%	11%	0%

**Table 2:** Experiment on DaSNet architectures and training methods (G stand for the GFPN) (${}^{*}$ F stands for fruit, B stands for branch).

Index	Model	Method	${\mathrm{AP}}_{50}$	$F_{1}$	${\mathrm{MIoU}}_{F}^{*}$	${\mathrm{MIoU}}_{B}^{*}$
1	DaSNet-A(G)	M1	0.792	0.796	0.849	0.683
2	DaSNet-B(G)	M1	0.803	0.8	0.857	0.703
3	DaSNet-C(G)	M1	0.819	0.819	0.86	0.76
4	DaSNet-B(G)	M2	**0.827**	0.821	0.865	0.757
5	DaSNet-C(G)	M2	0.823	0.824	0.864	0.762
6	DaSNet-B(FPN)	M2	0.799	0.792	0.832	0.722

**Table 3:** Time efficiency and weights size of developed model (tested on GTX1080Ti).

Index	Model	Inference Time	Weights Size
1	DaSNet-A(G)	**30** ms	9.6 M
2	DaSNet-B(G)	32 ms	12.8 M
3	DaSNet-C(G)	40 ms	15.8 M

**Table 4:** Experiment of prediction performance on validation set (640 × 480) with GTX1080Ti.

Index	Model	${\mathrm{AP}}_{50}$	$F_{1}$	Weights Size	Time
1	DaSNet-B(lw-net)	0.827	0.821	12.8 M	**32** ms
2	DaSNet-B(ResNet-50)	0.831	0.825	112 M	47 ms
3	DaSNet-B(ResNet-101)	**0.836**	0.832	188 M	72 ms
4	DaSNet-B(Darknet-53)	0.832	0.827	176 M	50 ms
5	YOLO-V3(Darknet-53) [31]	0.80	0.797	248 M	48 ms
6	YOLO-V3(Tiny) [31]	0.782	0.776	35.4 M	38 ms
7	Faster-RCNN (VGG-16) [22]	0.817	0.813	533 M	136 ms

**Table 5:** Experiment of semantic segmentation performance on validation set (640 × 480) with GTX1080Ti (${}^{*}$ F stands for the Fruit, while B stands for the branch).

Index	Model	${\mathrm{MIoU}}_{F}^{*}$	${\mathrm{MIoU}}_{B}^{*}$
1	DaSNet-B-Add(lw-net)	0.858	0.731
2	DaSNet-B-Concat(lw-net)	0.865	0.757
3	DaSNet-B-Concat(ResNet-50)	0.87	0.763
4	DaSNet-B-Concat(ResNet-101)	**0.876**	0.772
5	DaSNet-B-Concat(Darknet-53)	0.868	0.762
6	FCN-8s(ResNet-50) [36]	0.853	0.718
7	FCN-8s(ResNet-101) [36]	0.861	0.733
